# Asthma remission and its predictors in severe asthma: real-world study from the Korean severe asthma registry

**DOI:** 10.1186/s12931-025-03451-y

**Published:** 2025-12-27

**Authors:** Seung-Eun Lee, Byung-Keun Kim, Noeul Kang, Youngsoo Lee, Yoon-Seok Chang, Da Woon Sim, Hyo-In Rhyou, Jae-Woo Jung, Jae-Woo Kwon, Sujeong Kim, Taehoon Lee, Ga-Young Ban, Kyoung-Hee Sohn, Sang-Hoon Kim, An-Soo Jang, Sung-Yoon Kang, Min Suk Yang, So Ri Kim, Hyun Jung Jin, Young-Hee Nam, Ji Hyun Oh, Min-Hye Kim, Jin An, Hwa Young Lee, Han-Ki Park, Eun-Jung Jo, Ji-Ho Lee, Heung-Woo Park, Joo-Hee Kim, Woo-Jung Song, Sang-Heon Kim, So-Young Park

**Affiliations:** 1 Department of Internal Medicine, School of Medicine, Pusan National University, Pusan National University Yangsan Hospital, Yangsan, Korea; 2 Division of Pulmonology, Allergy and Critical Care Medicine, Department of Internal Medicine, Korea University College of Medicine, Seoul, Korea; 3 Division of Allergy, Department of Medicine, Samsung Medical Center, Sungkyunkwan University School of Medicine, Seoul, Korea; 4 Department of Allergy and Clinical Immunology, Ajou University School of Medicine, Suwon, Korea; 5 Department of Internal Medicine, Seoul National University Bundang Hospital, Seoul National University College of Medicine, Seongnam, Korea; 6 Department of Allergy and Clinical Immunology, Chonnam National University Hospital, Chonnam National University Medical School, Gwangju, Korea; 7 Department of Internal Medicine, Haeundae Paik Hospital, Inje University College of Medicine, Busan, South Korea; 8 Department of Internal Medicine, Chung-Ang University College of Medicine, Seoul, Korea; 9 Department of Internal Medicine, Division of Allergy and Clinical Immunology, Gangwon National University Hospital, Chuncheon, Korea; 10 Department of Internal Medicine, Kyungpook National University School of Medicine, Daegu, Korea; 11 Department of Internal Medicine, Division of Pulmonary, Allergy and Critical care medicine, Ulsan University Hospital, Ulsan, Korea; 12 Department of Internal Medicine, Division of Pulmonary, Allergy and Critical care medicine, Hallym University Gangdong Sacred Heart Hospital, Seoul, Korea; 13 Department of Internal Medicine, Division of Pulmonary, Allergy and Critical care medicine, Kyoung-Hee University Hospital, Seoul, Korea; 14 Department of Internal Medicine, Eulji University College of Medicine, Seoul, Korea; 15 Department of Internal Medicine, Division of Pulmonary, Allergy and Critical care medicine, Sunchunhyang University Bucheon Hospital, Bucheon, Korea; 16 Department of Internal Medicine, Gachon University Gil Medical Center, Incheon, Korea; 17 Department of Internal Medicine, Division of Allergy and Clinical Immunology, Seoul National University Boramae Hospital, Seoul, Korea; 18 Division of Respiratory Medicine and Allergy, Department of Internal Medicine, Chonbuk National University Medical School, Jeonju, Korea; 19 Department of Internal Medicine, Yeungnam University College of Medicine, Daegu, Korea; 20 Department of Internal Medicine, Dong-A University School of Medicine, Busan, Korea; 21 Division of Allergy and Clinical Immunology, Department of Internal Medicine, Kosin University College of Medicine, Busan, Korea; 22 Department of Internal Medicine, College of Medicine, Ewha Womans University, Seoul, Korea; 23 Department of Pulmonary, Allergy and Critical Care Medicine, Kyung Hee University Hospital at Gangdong, College of Medicine, Kyung Hee University, Seoul, Korea; 24 Division of Allergy, Department of Internal Medicine, Seoul St Mary’s Hospital, College of Medicine, the Catholic University of Korea, Seoul, Korea; 25 Division of Allergy and Clinical Immunology, Department of Internal Medicine, School of Medicine, Kyungpook National University, Kyungpook National University Chilgok Hospital, Daegu, Korea; 26 Department of Internal Medicine, Pusan National University College of Medicine, Busan, Korea; 27 Department of Internal Medicine, Yonsei University Wonju College of Medicine, Wonju, Korea; 28 Department of Internal Medicine, Seoul National University College of Medicine, Seoul, Korea; 29 Department of Internal Medicine, Division of Pulmonary Medicine, Hallym University Sacred Heart Hospital, Hallym University Medical School, Anyang, Republic of Korea; 30 Department of Allergy and Clinical Immunology, Asan Medical Center, University of Ulsan College of Medicine, Seoul, Korea; 31 Division of Pulmonary Medicine and Allergy, Department of Internal Medicine, Hanyang University College of Medicine, Seoul, Korea; 32 Division of Pulmonology, Allergy and Critical Care Medicine, Department of Internal Medicine, Chung-Ang University Gwangmyeong hospital, Gwangmyeong, Korea; 33 Department of Internal Medicine, Chung-Ang University Gwangmyeong Hospital, Chung-Ang University College of Medicine, 110, Deokan-ro, Gwangmyeong-si, Gyeonggi-do Republic of Korea

**Keywords:** Asthma, Severe asthma, Remission, Biologics, Chronic cough

## Abstract

**Background:**

Remission has emerged as a therapeutic goal in asthma, but most studies in severe asthma have focused on biologic-treated patients in controlled settings. Real-world data from Asian populations are scarce. We aimed to evaluate the achievement and predictors of asthma remission in Korean patients with severe asthma using a nationwide prospective cohort.

**Methods:**

We analyzed 405 patients with severe asthma from the Korean Severe Asthma Registry (KoSAR) who completed 12-month follow-up. Remission was classified at 12 and 24 months as complete clinical remission (CCR; ACT ≥ 20, no exacerbations, no oral corticosteroid [OCS] use, and FEV₁ ≥80% or improvement ≥ 100 mL), clinical remission (CR; first three criteria), partial remission (PR; ≥1 criterion), and no remission (NR; none). Ordinal logistic regression identified baseline predictors of higher remission.

**Results:**

At 12 months, CCR, CR, PR, and NR were achieved in 5.9%, 18.3%, 67.9%, and 7.9% of participants. Among those with 24-month follow-up (*n* = 139), remission status was largely stable. Higher baseline ACT score (OR: 1.19, 95% CI 1.12–1.27) predicted remission, while maintenance OCS use (OR: 0.11, 95% CI 0.05–0.25) and chronic cough (OR: 0.39, 95% CI 0.17–0.89) were negatively associated. Remission groups had better baseline lung function, fewer exacerbations, and low WBC counts. Baseline biologic use was more common in CCR, CR groups, whereas NR patients more frequently received methylxanthines, macrolides, and OCS.

**Conclusions:**

Clinical predictors, including asthma control, OCS use, and chronic cough may help guide remission-focused strategies in the treatment of severe asthma.

**Supplementary Information:**

The online version contains supplementary material available at 10.1186/s12931-025-03451-y.

## Introduction

Asthma is a chronic inflammatory airway disease with a variable clinical course [1]. Patients with severe asthma continually experience uncontrolled symptoms, frequent exacerbations, and impaired quality of life despite high-dose inhaled corticosteroids (ICS) and additional controller medications [1]. Managing this population remains a significant clinical challenge.

In recent years, the treatment goals for asthma have evolved beyond symptom control or lung function improvement to include achieving remission and minimizing future health risks [2–4]. Originally conceptualized in the management of other chronic inflammatory disorders, such as rheumatoid arthritis and inflammatory bowel disease, remission in asthma refers to the absence of symptoms and exacerbations, discontinuation of oral corticosteroids (OCS), and preservation of lung function [5, 6]. Achieving remission may offer substantial benefits, including improved prognosis, reduced medication burden, and more individualized treatment approaches [2, 4].

Currently, there is no universally accepted definition of asthma remission, and the criteria vary across studies [2, 7, 8]. Most existing research has focused on patients with mild-to-moderate asthma, or on patients with severe asthma in the context of biologic therapy evaluations [9–12]. Consequently, evidence specifically addressing the rates, clinical predictors, and longitudinal patterns of remission in patients with severe asthma in the real world is limited. Moreover, many previous studies have defined asthma remission using a binary framework and involved relatively short follow-up periods, which may limit their ability to fully characterize the variability and potential longitudinal stability of the remission status [2, 13, 14].

To address these gaps, this study aimed to evaluate asthma remission in a real-world cohort of patients with severe asthma enrolled in the Korean Severe Asthma Registry (KoSAR) [15]. We classified the patients into four categories of remission based on symptom control, exacerbation history, OCS use, and pulmonary function [8].

## Methods

### Patients and study design

This study was conducted using data from KoSAR, a nationwide, multicenter, prospective observational cohort organized and operated supported by the Severe Asthma Working Group of the Korean Academy of Asthma, Allergy, and Clinical Immunology (KAAACI) [15]. The registry enrolled patients from 41 tertiary or secondary referral centers across South Korea. Patients are eligible for inclusion in KoSAR if they are diagnosed with severe asthma (SA) based on the following criteria: (1) uncontrolled asthma despite treatment for more than one year at GINA step 4 or 5; or (2) well-controlled asthma on step 4 or 5 treatment but with a history of ≥ 1 unscheduled urgent care visit, ≥ 3 courses of systemic corticosteroids in the past year, a near-fatal asthma exacerbation, or asthma deterioration upon tapering ICS or OCS by 25% or more [15].

For this analysis, 645 patients enrolled between April 2022 and August 2024 were initially screened. Of these, 405 patients were included in the final study population based on the availability of baseline data and complete 12-month follow-up, sufficient to assess asthma remission status. Clinical variables such as symptom scores, exacerbation history, laboratory findings, and pulmonary function were repeatedly collected every 6 months throughout the follow-up period. Comorbidities were identified based on physician-diagnosed history documented in medical records. Written informed consent was obtained from all patients. The study protocol was approved by the Institutional Review Boards of all participating centers and was conducted in accordance with the Declaration of Helsinki.

### Definition of remission

Remission status was classified at 12 months based on 4 clinical criteria: (1) well-controlled symptoms, defined as an Asthma Control Test (ACT) score ≥ 20; (2) no systemic corticosteroid use during the past 12 months; (3) absence of moderate-to-severe exacerbations in the past year; and (4) stable or improved lung function, defined as either pre-bronchodilator forced expiratory volume in 1 s (FEV₁) ≥ 80% predicted or an increase in FEV₁ of ≥ 100 mL from baseline [8].

Subjects were then categorized into one of the four remission groups based on the number and combination of criteria met. Those who satisfied all four criteria were considered to have complete clinical remission (CCR), whereas those who met criteria 1, 2, and 3 regardless of lung function were classified as having clinical remission (CR). Patients not meeting CCR or CR but fulfilling ≥ 1 criterion were classified as partial remission (PR). Those who did not meet any of the criteria were categorized as having no remission (NR). This definition was adapted from prior consensus criteria to fit the real-world data and longitudinal design of our cohort [7, 8, 16, 17].

### Statistical analysis

Continuous variables were compared using ANOVA or Kruskal-Wallis tests, and categorical variables with chi-squared test. Results are presented as mean ± standard deviation (SD) or median (interquartile range, IQR). Statistical significance was defined as a two-sided p-value of < 0.05.

To identify the baseline clinical predictors of asthma remission, an ordinal logistic regression analysis was performed using a proportional odds model. The outcome variable was the 12-month remission status, which was categorized into four ordered groups based on clinical and functional criteria: (1) NR; (2) PR; (3) CR; and (4) CCR. The following baseline variables were included as candidate predictors: ACT score, use of maintenance OCS, number of moderate-to-severe exacerbations in the previous year, use of biologics, blood eosinophil count, FEV % predicted value, and presence of chronic cough. These baseline variables were selected based on their significant differences among the remission groups in univariate comparisons. All the predictors were simultaneously entered into the model. Continuous variables were analyzed per unit increase (e.g., per 1-point increase in ACT score). Binary variables such as OCS use and chronic cough were coded as 1 for ‘yes’ and 0 for ‘no.’ Odds ratios (ORs) and the corresponding 95% confidence intervals (CIs) were calculated. An OR > 1 indicated an increased probability of achieving higher remission. The model performance and proportional odds assumptions were evaluated using standard diagnostics. A forest plot was generated to display the ORs and CIs for each predictor.

All analyses were conducted using R software (R Project for Statistical Computing, Vienna, Austria; www.r-project.org/). Statistical significance was set at a two-sided *p*-value < 0.05.

## Results

### Asthma remission status at 12 and 24 months

**Fig. 1:**
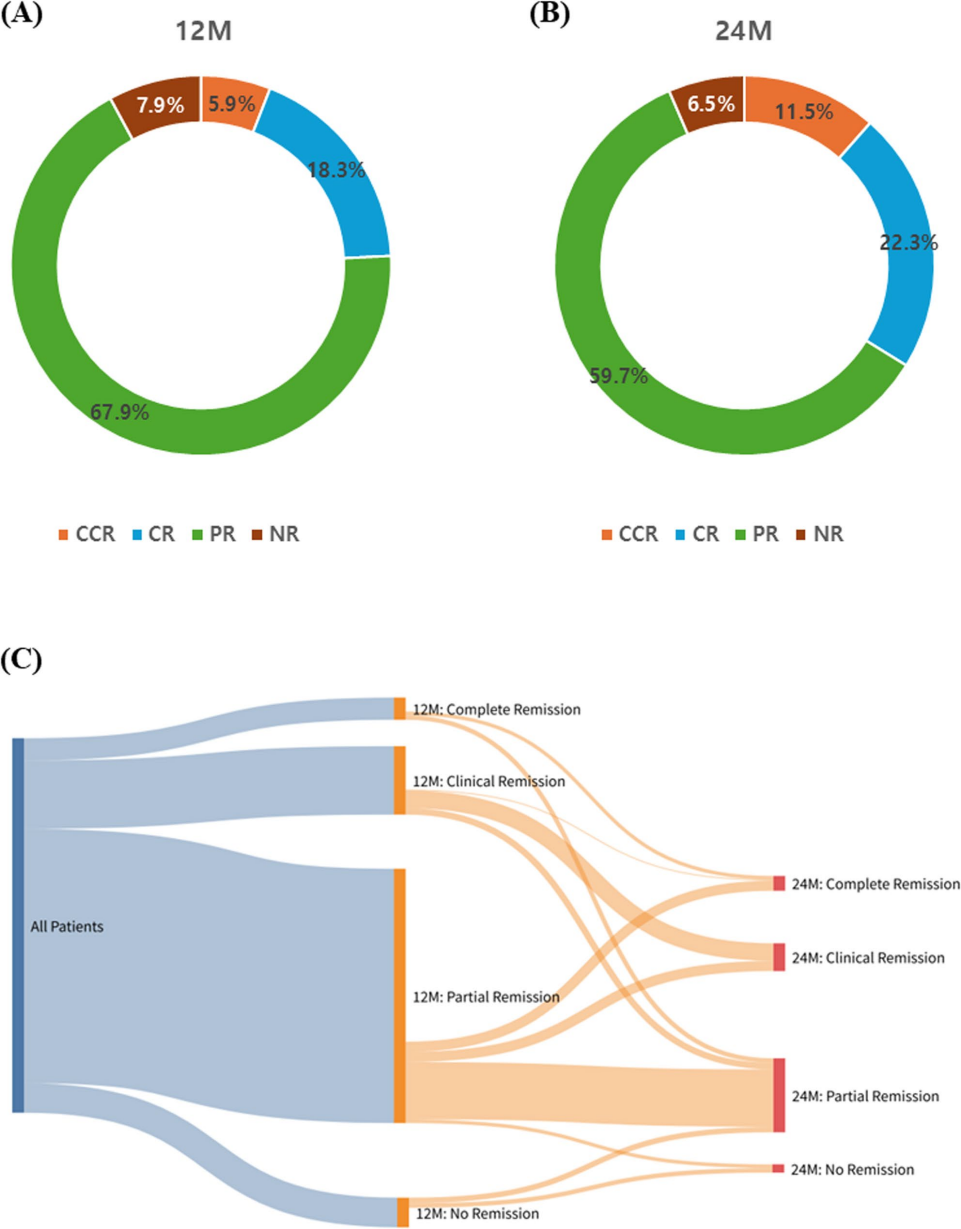
Distribution of asthma remission status at 12 and 24 months. Pie charts show the proportion of each group at (**A**) 12 months and (**B**) 24 months. (**C**) Sankey plot showing the longitudinal transition in remission status between 12 and 24 months. Each flow represents the number of patients moving from a specific remission group at 12 months to a corresponding group at 24 months

At 12 months after enrollment, achievement of remission of the study subjects (*N* = 405) was as follows: CCR, 24 patients (5.9%); CR, 74 patients (18.3%); PR, 275 patients (67.9%); and no NR, 32 patients (7.9%) **(**Fig. 1A**)**. By 24 months, among patients with complete follow-up data (*N* = 139), CCR was achieved in 16 (11.5%), CR in 31 (22.3%), PR in 83 (59.7%), and NR in nine (6.5%) patients (Fig. 1B). Compared to the 12-month distribution, the proportion of patients achieving CCR or CR increased slightly at 24 months, rising from 24.2% to 33.8%. Longitudinal transitions in the remission status between the 12- and 24-month time points are shown in Fig. 1C. Among patients with complete 24-month follow-up, 4 remained in complete clinical remission (CCR → CCR), and 19 maintained clinical remission (CR → CR). Notably, no patients transitioned from CCR or CR to no remission (NR), suggesting relative stability of remission status over time. Sankey plots revealed notable trends in remission stability and group transitions. Sankey plots revealed notable trends in remission stability and group transitions.

### Baseline clinical characteristics by remission group

Baseline clinical characteristics of the remission group at 12 months are summarized in Table 1. There were no statistically significant differences among the groups in terms of age, sex, age at asthma onset, or smoking status. Similarly, atopy prevalence and distribution of the GINA steps were also comparable across groups. In contrast, indicators of asthma control and quality of life at baseline showed marked differences across the remission status. Patients in the CCR and CR groups had significantly better ACT, SAQ, and EQ-VAS scores than those in the PR and NR groups (*p* < 0.001 for all). Notably, the NR group had a significantly higher number of moderate-to-severe exacerbations in the prior 12 months (median 4.0) than other groups, with a clear trend across the remission categories (*p* < 0.001). The total number of lifetime exacerbations was also the highest in the NR group (*p* = 0.003).

**Table 1:** Baseline characteristics (*N* = 405)

	CCR (*N* = 24)	CR (*N* = 74)	PR (*N* = 275)	NR (*N* = 32)	*P*-value
**Age**,** y**	55.7 ± 10.3	58.3 ± 11.8	56.9 ± 13.1	52.8 ± 15.1	0.269
**Sex**,** female N(%)**	16(66.7)	32(43.2)	163(59.3)	18(56.2)	0.067
**BMI**,** kg/m**^**2**^	24.1(22.2–25.8)	25.3(23.3–27.4)	24.4(22.3–27.4)	23.7(22.3–25.4)	0.221
**Asthma onset age**,** y**	42.6 ± 13.0	44.9 ± 14.3	42.8 ± 15.8	41.3 ± 17.0	0.581
**Smoking status**,** N(%)**					0.275
**Never smoker**	15(62.5)	35(47.3)	170(61.8)	15(46.9)	
**Ex-smoker**	8(33.3)	31(41.9)	84(30.5)	13(40.6)	
**Current smoker**	1(4.2)	8(10.8)	21(7.6)	4(12.5)	
**Insurance type**,** N(%)**	(*n* = 16)	(*n* = 61)	(*n* = 204)	(*n* = 24)	0.0602
**National health**	14(87.5)	58(95.1)	172(84.3)	17(70.8)	
**Medical aid type 1**	2(12.5)	3(4.9)	17(8.3)	6(25.0)	
**Medical aid type 2**	0(0.0)	0(0.0)	8(3.9)	0(4.2)	
**Others**	0(0.0)	0(0.0)	7(3.4)	0(0.0)	
**Atopy**,** N(%)**	14(58.3)	31(41.9)	137(49.8)	12(37.5)	0.301
**GINA step**,** N(%)**					0.163
**Step 4**	7(29.2)	33(44.6)	87(31.6)	9(28.1)	
**Step 5**	17(70.8)	41(55.4)	188(68.4)	12(71.9)	
**Asthma exacerbation**,** n**					
**Total**	7.8 ± 21.0 (*n* = 24) 1.0(0.0–3.5.0.5)	7.5 ± 27.7 (*n* = 70) 2.0(0.0–4.8.0.8)	6.1 ± 20.9 (*n* = 268) 2.0(0.0–5.0)	14.6 ± 22.8 (*n* = 31) 4.0(2.0–17.5.0.5)	**0.003**
**Previous 1 year**	0.2 ± 0.7 0.0(0.0–0.0)	0.5 ± 1.7 0.0(0.0–0.0)	1.0 ± 2.6 0.0(0.0–1.0)	3.3 ± 6.3 1.0(0.0–4.3.0.3)	**< 0.001**
**ACT score**	23.0(20.0–24.0)	22.0(20.0–24.0)	19.0(15.0–22.0)	15.0(11.0–18.3.0.3)	**< 0.001**
**SAQ score**	96.5(90.0–105.8.0.8)	99.5(86.0–109.0.0.0)	88.0(71.5–100.0)	74.5(58.3–91.5)	**< 0.001**
**EQ-VAS**	70.0(67.5–80.0)	70.0(52.5–80.0)	60.0(50.0–80.0)	50(38.8–60.0)	**< 0.001**
**Total IgE**,** IU/L**	374.0(221.6–803.0) (*n* = 21)	200.0(105.4–557.7.4.7) (*n* = 63)	251.0(73.5–566.5.5.5) (*n* = 235)	173.5(69.2–415.1.2.1) (*n* = 28)	0.124
**WBC**,** 10**^**3**^**/mm**^**3**^	6.6(5.5–8.5) (*n* = 23)	7.1(5.8–8.9) (*n* = 67)	7.6(6.3–9.3) (*n* = 243)	8.4(7.3–11.6) (*n* = 30)	**0.005**
**Eosinophil**,** %**	6.5(3.1–12.6) (*n* = 23)	5.1(2.7–10.8) (*n* = 66)	5.6(2.1–9.9) (*n* = 239)	3.5(1.8–10.3)(*n* = 30)	0.624
**Eosinophil count**,**/mm**^**3**^	403.2(76.5–560.0) (*n* = 23)	280.3(57.5–622.5.5.5) (*n* = 66)	274.4(93.3–622.5.3.5) (*n* = 240)	230.0(87.0–630.0.0.0) (*n* = 30)	0.912
**CRP**,** mg/dL**	0.2(0.1–0.4) (*n* = 14)	0.3(0.1–1.0.1.0) (*n* = 29)	0.2(0.1–0.5) (*n* = 113)	0.2(0.1–1.1) (*n* = 15)	0.209
**Sputum Eosinophil**,** %**	30.5(8.0–55.7.0.7) (*n* = 12)	33.0(13.0–53.0) (*n* = 23)	23.0(3.0–55.0) (*n* = 61)	6.0(3.0–30.0) (*n* = 3)	0.432
**FeNO**,** ppb**	70.0(50.3–105.8.3.8) (*n* = 16)	47.5(30.3–67.0) (*n* = 44)	43.0(24.0–80.0) (*n* = 171)	34.5(23.3–68.5) (*n* = 26)	**0.029**
**Pre-BD FVC**,** L**	3.4(2.7–3.9)	3.0(2.6–3.8)	2.9(2.3–3.5)	2.7(2.4–3.2)	0.068
**Pre-BD FVC**,** %**	92.2(81.1–98.0)	77.9(68.4–86.6)	78.7(68.3–88.6)	73.6(66.8–80.7)	**0.004**
**Post-BD FVC**,** L**	3.3(3.0–3.8.0.8) (*n* = 14)	3.3(2.7–4.0.7.0) (*n* = 43)	3.0(2.4–3.6) (*n* = 168)	2.8(2.3–3.5) (*n* = 18)	0.169
**Post-BD FVC**,** %**	92.4(85.1–95.2) (*n* = 14)	78.1(70.7–89.2) (*n* = 43)	80.9(70.9–91.6) (*n* = 168)	75.4(67.3–82.8) (*n* = 18)	0.071
**Pre-BD FEV**_**1**_, **L**	2.5(1.7–2.7)	1.9(1.6–2.7)	1.8(1.5–2.4)	1.8(1.3–2.2)	**0.011**
**Pre-BD FEV**_**1**_, **%**	82.8(67.6–89.4)	67.0(59.0–75.4.0.4)	68.0(53.6–77.7)	59.1(45.2–68.0)	**< 0.001**
**Post-BD FEV**_**1**_, **L**	2.6(2.1–2.7) (*n* = 14)	2.0(1.6–2.9) (*n* = 43)	1.9(1.6–2.5) (*n* = 168)	1.7 (1.0–2.2.0.2) (*n* = 18)	**0.048**
**Post-BD FEV**_**1**_, **%**	83.7(65.2–90.0) (*n* = 14)	72.0(65.3–79.4) (*n* = 43)	70.5(58.6–81.7) (*n* = 168)	58.4(46.1–68.1) (*n* = 18)	**0.009**
**FEV1 change**,** %**	4.8 ± 12.6	5.4 ± 6.1	7.9 ± 12.8	6.0 ± 9.0	0.070
**Pre-BD FEV**_**1**_**/FVC**,** %**	74.0(63.8–77.3)	67.9(59.8–73.6)	68.0(57.0–76.0)	63.8(55.3–76.0)	0.141
**Post-BD FEV**_**1**_**/FVC**,** %**	74.5(64.3–79.5) (*n* = 14)	69.0(63.0–76.5.0.5) (*n* = 43)	71.0(59.0–77.8.0.8) (*n* = 168)	63.0(45.0–80.8.0.8) (*n* = 18)	0.432
**Pre-BD FEF**_**25 − 75**_,**%**	1.7(1.1–2.3)	1.1(0.7–1.8)	1.2(0.6–1.8)	1.0(0.7–1.6)	0.148
**Post-BD FEF**_**25 − 75**_,**%**	1.8(1.1–2.2) (*n* = 14)	1.2(0.9–1.8) (*n* = 43)	1.2(0.6–2.1) (*n* = 159)	1.1 (0.4–1.8) (*n* = 18)	0.234

*CCR* Complete clinical remission, *CR* Clinical remission, *PR* Partial remission, *NR* no remission, *BMI* Body Mass Index, GINA, *ACT* Asthma Control Test, *SAQ* Severe Asthma Questionnaire, *EQ-VAS* EuroQol Visual Analogue Scale, *IgE* Immunoglobulin E, *IU* International unit, *WBC* White blood cell, *CRP* C-reactive protein, *FeNO* Fractional exhaled nitric oxide, *BD* bronchodilator, *FVC* Forced vital capacity, *FEV*_1_, Forced expiratory volume in 1 s; *FEF25*, forced expiratory flow at 25–75%

Regarding laboratory parameters, the total white blood cell (WBC) count was highest in the NR group and lowest in the CCR group (*p* = 0.005). Although blood eosinophil counts tended to be higher in the CCR group, the difference was not statistically significant (*p* = 0.912). In contrast, the FeNO levels were significantly higher in the CCR group than in the other groups (*p* = 0.029).

Pulmonary function tests revealed significant differences between the groups. Overall, better baseline lung function was associated with a greater likelihood of achieving CCR or CR at 12 months. Pre-bronchodilator forced vital capacity (FVC)% and FEV₁ % predicted value were highest in the CCR group and lowest in the NR group (median 92.2% vs. 73.6%, *p* = 0.004, 82.8% vs. 59.1%, *p* < 0.001 respectively). Similar trends were observed for post-bronchodilator FEV₁, FVC, and FEV₁/FVC, though not all reached statistical significance.

### Comorbidities across remission groups

Comorbidities at baseline were compared across the remission groups to assess whether specific conditions were associated with asthma remission status (Supplementary Table 1).

Allergic or systemic comorbidities, including allergic rhinitis, chronic rhinosinusitis, nasal polyps, and atopic dermatitis, were similarly distributed across the groups, with no statistically significant differences (*p* > 0.05). Systemic comorbidities, such as diabetes mellitus, hypertension, and osteoporosis, were more prevalent in the NR group (18.8%, 40.6%, and 34.4%, respectively) than in the CCR group (8.3%, 25.0%, and 12.5%, respectively), although these differences were not statistically significant (*p* = 0.452, *p* = 0.362, and *p* = 0.137, respectively). Cardiovascular conditions, including myocardial infarction or angina, heart failure, and arrhythmia, were relatively uncommon in this cohort and did not differ significantly across the groups. Similarly, gastrointestinal disorders such as GERD and peptic ulcer disease showed no significant differences. In terms of neuropsychiatric conditions, anxiety and depression were present in a small proportion of the patients, with a slightly higher prevalence in the NR group, although the difference was not statistically significant. However, chronic cough has emerged as a distinguishing feature. It was significantly more common in the NR group (28.1%) than in the CCR (12.5%), CR (5.4%), and PR (10.9%) groups (*p* = 0.009).

### Baseline medication usage by remission group

Medication use at baseline across the remission groups is summarized in Table 2. While the overall inhaler prescription patterns were similar between the groups, triple combination therapy (ICS/LABA/LAMA) was more frequently used in the NR group than in the other groups, suggesting a greater treatment intensity in patients with poorer asthma control. LTRA use was consistent across all groups, with approximately 80% of patients receiving LTRA irrespective of remission status (*p* = 0.853). In contrast, the use of methylxanthines and macrolides was significantly more common in the NR group (*p* = 0.001 and *p* = 0.034, respectively), potentially reflecting the use of alternative or adjunct therapeutic strategies for difficult-to-treat patients. Although the ICS dose tended to be higher in the NR group, the difference was not statistically significant (*p* = 0.189). However, maintenance OCS use showed a marked difference across groups, with 75.0% of NR patients receiving OCS at baseline compared with only 4.2% in the CCR group (*p* < 0.001). Among the patients who achieved CCR at 12 months, 50.0% were receiving biologics, whereas only 18.8% of patients in the NR group were treated with biologics (*p* = 0.042).

**Table 2:** Medication (*N* = 399)

	CCR (*N* = 24)	CR (*N* = 74)	PR (*N* = 275)	NR (*N* = 32)	*P*-value
**Inhaler type**					0.065
**ICS/LABA**,** N(%)**	14(58.3)	35(47.3)	105(38.2)	9(28.1)	
**ICS/LABA/LAMA**,** N(%)**	10(41.7)	39(52.7)	170(61.8)	23(71.9)	
**LTRA**,** N(%)**	21(87.5)	57(77.0)	221(80.4)	28(87.5)	0.507
**Methylxanthine**,** N(%)**	5(20.8)	9(12.2)	89(32.4)	15(46.9)	**0.001**
**Macrolide**,** N(%)**	0(0)	3(4.1)	25(9.1)	6(18.8)	**0.034**
ICS dose*,** mcg/day**	520(290–640)	500(400–920)	400(400–680)	620(400–960)	0.638
**OCS maintenance**,** N(%)**	1(4.2)	4(5.4)	54(19.6)	24(75.0)	**< 0.001**
**OCS maintenance dose**,** mg/day**	10.5	3.8(2.2–11.2)	10.0(5.0–26.9.0.9)	10.0(5.0–15.0)	0.572
**Biologics**,** N(%)**	12(50.0)	17(23.0)	73(26.5)	6(18.8)	**0.042**
**Mepolizumab**,** N**	0	0	6	1	
**Reslizumab**,** N**	4	4	22	2	
**Benralizumab**,** N**	1	2	4	0	
**Dupilumab**,** N**	6	5	21	2	
**Omalizumab**,** N**	1	6	20	1	

* Budesonide equivalent dose

*CCR* Complete clinical remission, *CR* Clinical remission, *PR* Partial remission, *NR* No remission, *ICS* Inhaled corticosteroids, *LABA* Long- acting beta2agonist, *LAMA* Long-acting muscarinic actagonist, *LTRA* Leukotriene receptor antagonist, *OCS* Oral corticosteroids

#### Characteristics of biologic users by remission status

A subgroup analysis was performed for patients treated with biological agents (Table 3). Among the 103 patients who received biologics at enrollment, 29 (28.2%) achieved CCR or CR, while 6 (5.8%) remained in the NR group at 12 months despite advanced therapy. Patients in the NR group exhibited distinct clinical features even within the biologically treated population. Patients in the CCR/CR group were younger (median 50.0 years, IQR 47.0–57.0) compared to those in the NR group (59.5 years, IQR 51.5–75.0), though the difference was not statistically significant (*p* = 0.196). The proportion of female patients was comparable between the two groups (44.8% vs. 66.7%, *p* = 0.599). Although blood eosinophil counts were numerically higher in the remission group [360/mm³ (IQR 47.7–1090.0)] compared to the NR group [91/mm³ (IQR 21.6–174.0)], this difference was not statistically significant (*p* = 0.515). Importantly, the OCS maintenance rate was significantly lower in the CCR/CR group (27.6%) than in the NR group (83.3%, *p* < 0.001), indicating that systemic steroid dependence may be associated with failure to achieve remission, even under biologic therapy. Similarly, the number of asthma exacerbations in the past 12 months was markedly different, with a median of 0 (IQR 0.0–0.0) in the CCR/CR group and 6.0 (IQR 2.0–7.8) in the NR group (*p* < 0.001). Baseline pre-bronchodilator FEV₁ % predicted was significantly lower in the NR group (median 61.9%, IQR 55.2–66.6) than in the CCR/CR group (74.8%, IQR 66.4–88.4; *p* = 0.044).

**Table 3:** Comparison of clinical characteristics between Biologics-Treated patients with and without remission at 12 months

Clinical Variable	CCR/CR (with remission, *N* = 29)	NR (without remission, *N* = 6)	*p*-value
**Age**,** y**	50.0(47.0–57.0)	59.5(51.5–75.0)	0.196
**Sex Female**,** N(%)**	13(44.8)	4(66.7)	0.599
**Blood Eosinophil count**,**/mm**^**3**^	360(47.7–1090.0)	91(21.6–174.0)	0.515
**OCS maintenance**,** N(%)**	8(27.6)	5(83.3)	**< 0.001**
**Asthma exacerbation in past 12 M**,** n**	0(0.0–0.0)	6.0(2.0–7.8.0.8)	**< 0.001**
**Pre-BD FEV**_**1**_, **%**	74.8(66.4–88.4)	61.9(55.2–66.6)	**0.044**

*CCR* Complete clinical remission, *CR* Clinical remission, *NR* No remission, *BD* Bronchodilator, *OCS* Oral corticosteroids, *FEV*_1_ Forced expiratory volume in 1s

### Predictors of asthma remission at 12 months

**Fig. 2:**
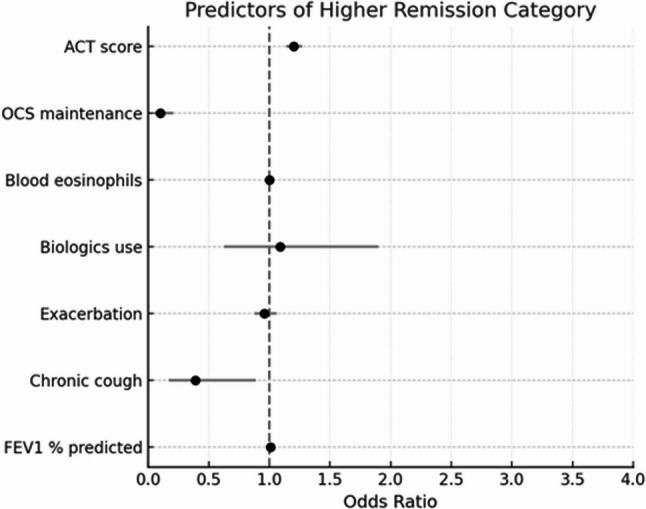
Forest plot of ordinal logistic regression showing predictors of higher asthma remission category (No → Partial → Clinical → Complete Clinical remission). Odds ratios (log scale) and 95% confidence intervals are displayed. ACT score and absence of OCS maintenance therapy were significantly associated with better remission status. Chronic cough was also associated with lower odds of achieving higher remission ACT, Asthma Control Test; OCS, oral corticosteroid

Ordinal logistic regression was performed to identify the baseline clinical predictors associated with increasing levels of asthma remission at 12 months, defined across four ordered categories: NR, PR, CR, and CCR. In this model, an odds ratio (OR) greater than 1 indicated an increased likelihood of achieving a higher remission category (e.g., from partial to clinical remission), whereas an OR less than 1 reflected a reduced probability of remission progression. Among the tested variables, a higher ACT score was independently associated with greater odds of achieving a higher remission category (OR: 1.19, 95% CI: 1.12–1.27, *p* < 0.001). Maintenance OCS therapy during the past 6 months was significantly associated with lower remission levels (OR: 0.11, 95% CI: 0.05–0.25, *p* < 0.001). Similarly, the presence of chronic cough at baseline was associated with a reduced likelihood of remission (OR: 0.39, 95% CI: 0.17–0.89, *p* = 0.026). Other variables, including the number of moderate-to-severe exacerbations in the prior year (OR: 0.96, 95% CI: 0.88–1.06, *p* = 0.416), use of biologics (OR: 1.09, 95% CI: 0.63–1.90, *p* = 0.756), blood eosinophil count (OR: 1.00, 95% CI: 1.00–1.00, *p* = 0.290), and baseline FEV₁ % predicted (OR: 1.01, 95% CI: 0.99–1.02, *p* = 0.381), were not statistically significant predictors in this model. A forest plot of the odds ratios and 95% confidence intervals is shown in Fig. 2, and the full regression results are summarized in Table 4.

**Table 4:** Predictors of higher remission category (Ordinal logistic Regression)

Clinical Variable	Odds Ratio	95% Confidence Interval	*p*-value
**ACT score**	1.19	1.12–1.27	**< 0.001**
**OCS maintenance**	0.11	0.05–0.25	**< 0.001**
**Blood Eosinophil count**,**/mm**^**3**^	1.00	1.00–1.00	0.290
**Biologics use**	1.09	0.63–1.90	0.756
**Asthma exacerbation in past 12M**	0.96	0.88–1.06	0.416
**Chronic cough**	0.39	0.17–0.89	**0.026**
**FEV₁ % predicted**	1.01	0.99–1.02	0.381

*ACT* Asthma Control Test, OCS, oral corticosteroids, FEV_1_, forced expiratory volume in 1s

## Discussion

This study provides novel insights into the long-term control of severe asthma in a real-world clinical setting, and lays foundation for integrating remission-targeted strategies into routine clinical care and personalized asthma management. To the best of our knowledge, this is the first nationwide analysis of remission in Korea. At 12 months, CCR or CR was achieved in approximately 24% of patients and remission status was generally stable over time. Notably, patients who achieved CCR or CR at 12 months did not regress to NR by 24 months; 23 out of 33 (69.7%) maintained the same status. These findings highlight the stability of remission in severe asthma, even in real-world settings. Once high-level remission is achieved, it is usually sustained, and most patients remained in their initial group or moved only to adjacent categories, supporting its value as a prognostic marker. This is consistent with prior studies, including a retrospective analysis of mild-to-moderate asthma in which 80–89% of patients maintained remission over 10 years [9].

The clinical characteristics of the patients who achieved remission were consistent with previous studies [10, 16, 17]. Patients with higher baseline ACT, SAQ, and EQ-VAS scores, fewer exacerbations, and preserved lung function were more likely to achieve asthma remission at 12 months. This is consistent with previous findings that patients with lower baseline disease severity, better-preserved lung function, and well-controlled asthma are more likely to achieve remission [2, 17–19]. In our study, remission groups also demonstrated lower levels of systemic inflammatory markers, such as white blood cell count. However, CCR patients showed higher FeNO levels and blood eosinophil counts, suggesting a link between type 2 (T2) inflammation and responsiveness to biologics. While it remains unclear whether these inflammatory markers themselves directly contribute to remission or whether their association is mediated by the increased use of targeted biologics, these findings highlight the complex relationship between T2 inflammation and remission. Similar findings in the REDES study support the influence of T2 inflammation on remission [17].

Among comorbidities, chronic cough was the most strongly associated with failure to achieve asthma remission. Recent evidence has suggested that chronic cough is closely associated with poorly controlled or refractory asthma phenotypes [20, 21]. In studies exploring patients’ experiences with severe asthma, cough has been reported as one of the most distressing and disruptive symptoms [22–24]. The impact of cough on quality of life is independent of other parameters of severe asthma, including T2 inflammation, lung function, and comorbidities [25]. This highlights the need to address cough as a distinct therapeutic target in the management of severe asthma.

The mechanisms underlying cough in asthma are considered to be diverse, including T2 inflammation, comorbid conditions associated with cough, and cough reflex hypersensitivity [20, 26]. Among these, the role of T2 inflammation appears to be only partially related to asthma-associated cough [27]. In particular, in patients receiving maximal asthma therapy, persistent cough is more likely to be driven by heightened cough reflex sensitivity rather than by residual airway inflammation [28]. This highlights the need to address treatable traits beyond conventional asthma targets, such as cough hypersensitivity, when aiming for remission in patients with asthma and chronic cough. Furthermore, as current definitions of asthma treatment outcomes are primarily based on traditional pathophysiological paradigms, there is a growing need to redefine therapeutic goals in asthma to encompass cough-specific domains.

Consistent with this, our findings indicate that patients with chronic cough were significantly less likely to achieve remission. These results highlight the importance of identifying patients with asthma and coexisting chronic cough as this subgroup may require a more personalized and targeted treatment approach to improve long-term outcomes.

In terms of medication use, patients achieving remission were more likely to receive biologics, while those in the NR group more often used macrolides, methylxanthines, and maintenance OCS. Although biologics are known to improve control and remission, some patients remained in the NR group despite advanced therapy [10, 29, 30]. These patients typically had lower baseline lung function, higher OCS use, and more frequent exacerbations, reflecting multifactorial barriers to remission. The frequent use of macrolides and methylxanthines in the NR group may also reflect their use as adjunctive therapies in patients with refractory symptoms or persistent exacerbations despite receiving standard treatment [31–33]. Similarly, patients requiring OCS maintenance are likely to have poorly controlled asthma [34, 35]. In our study, such patients were significantly overrepresented in the NR group, suggesting not only a lack of disease control but also the possibility of persistent steroid dependency. This finding supports the interpretation that OCS dependence may serve as a clinical marker of poor prognosis and a major barrier to achieving remission in severe asthma.

Currently, there is no universally accepted definition of asthma remission, and various criteria have been proposed in recent literature, often incorporating components such as symptom control, absence of exacerbations, cessation of OCS, and preserved lung function [2, 3, 7, 36]. In this study, we adopted a pragmatic clinical definition (ACT score, exacerbation history, OCS use, lung function) to reflect real-world practice. However, our definition did not incorporate inflammatory markers of disease activity, such as blood eosinophils or FeNO, or radiological indicators of airway remodeling (e.g., chest CT), which are increasingly emphasized in the emerging concept of inflammatory remission. Consequently, some patients with subclinical or persistent T2 inflammation may have been misclassified as being in remission despite ongoing airway inflammation. This is a limitation of our study and highlights the need for future research that incorporates multidimensional definitions of remission that reflect both clinical and biological disease activities. In addition, this study had several other limitations. First, only a subset completed 24-month follow-up, potentially introducing selection bias and limiting generalizability. However, this reduction was primarily due to the timing of data extraction rather than true loss to follow-up, as many patients had not yet reached the 24-month visit at the time of analysis. Second, although we assessed a range of clinical and physiological parameters, we could not account for confounders such as psychosocial influences, inhaler technique, and environmental exposures, all known to affect asthma control and outcomes.

Nevertheless, this study had several strengths. This is the first nationwide investigation of asthma remission among patients with severe asthma in Korea using the largest nationwide real-world cohort. The KoSAR registry includes a wide range of patients, including older adults, those with multiple comorbidities, and individuals with persistent steroid dependence, thus closely reflecting the complexity of patients observed in routine clinical practice. Furthermore, the use of longitudinal data allowed us to assess the stability and prognostic value of the remission status over time, highlighting that patients who achieved complete or clinical remission at 12 months tended to maintain their status at 24 months. These findings underscore the clinical utility of remission assessment in patients with severe asthma and support the need for further research on personalized management strategies that can help patients achieve and sustain remission.

## Supplementary Information

Supplementary Material 1

## Data Availability

The datasets generated and/or analyzed during the current study are not publicly available due to patient privacy but are available from the corresponding author on reasonable request.

## Abbreviations

ACT: Asthma Control Test
CCR: Complete Clinical Remission
CR: Clinical Remission
PR: Partial Remission
NR: No Remission
OCS: Oral Corticosteroids
ICS: Inhaled Corticosteroids
FEV1: Forced Expiratory Volume in 1 Second
FVC: Forced Vital Capacity
GINA: Global Initiative for Asthma
KoSAR: Korean Severe Asthma Registry
T2: Type 2 (Inflammation)
WBC: White Blood Cell
SAQ: Severe Asthma Questionnaire
EQ-VAS: EuroQol Visual Analogue Scale
OR: Odds Ratio
CI: Confidence Interval
