# Supplementary material for: Asthma remission and its predictors in severe asthma: real-world study from the Korean severe asthma registry
Source: Respir Res. 2025 Dec 27;27:8. doi: 10.1186/s12931-025-03451-y (PMC12781308; doi:10.1186/s12931-025-03451-y)
Supplement: Supplementary file 1 — Supplementary Material 1 [file 12931_2025_3451_MOESM1_ESM.docx]

**Supplementary Table I. Comorbidities distribution by remission group**

| **Comorbidity N, %** | **C-CR**  **(N=24)** | **CR**  **(N=74)** | **PR**  **(N=275)** | **NR**  **(N=32)** | **P-value** |
| --- | --- | --- | --- | --- | --- |
| **Allergic rhinitis** | 17(70.8) | 48(64.9) | 175(63.6) | 18(56.2) | 0.720 |
| **Allergic conjunctivitis** | 3(12.5) | 1(1.4) | 19(6.9) | 4(12.5) | 0.092 |
| **Chronic rhinosinusitis** | 11(45.8) | 23(31.1) | 97(35.3) | 12(37.5) | 0.613 |
| **Nasal polyp** | 6(25.0) | 8(10.8) | 35(12.7) | 7(21.9) | 0.165 |
| **Atopic dermatitis** | 0(0.0) | 5(6.8) | 20(7.3) | 3(9.4) | 0.544 |
| **Chronic urticaria** | 1(4.2) | 2(2.7) | 32(11.6) | 2(6.2) | 0.079 |
| **Drug allergy** | 3(12.5) | 9(12.2) | 30(10.9) | 2(6.2) | 0.826 |
| **Food allergy** | 0(0.0) | 2(2.7) | 20(7.3) | 2(6.2) | 0.285 |
| **DM** | 1(4.2) | 9(12.2) | 39(14.2) | 9(28.1) | 0.064 |
| **HTN** | 10(41.7) | 25(33.8) | 82(29.8) | 8(25.0) | 0.519 |
| **MI, Angina** | 1(4.2) | 3(4.1) | 19(6.9) | 4(12.5) | 0.419 |
| **Heart failure** | 0(0.0) | 2(2.7) | 4(1.5) | 0(0.0) | 0.659 |
| **Arrhythmia** | 1(4.2) | 2(2.7) | 6(2.2) | 0(0.0) | 0.747 |
| **GERD** | 9(37.5) | 20(27.0) | 66(24.0) | 6(18.8) | 0.395 |
| **Osteoporosis** | 6(25.0) | 5(6.8) | 35(12.7) | 4(12.5) | 0.126 |
| **Fracture** | 1(4.2) | 7(9.5) | 23(8.4) | 3(9.4) | 0.872 |
| **Rheumatic disease** | 3(12.5) | 5(6.8) | 10(3.6) | 2(6.2) | 0.375 |
| **Depression** | 0(0.0) | 4(5.4) | 24(8.7) | 2(6.2) | 0.375) |
| **Sleep apnea** | 2(8.3) | 3(4.1) | 7(2.5) | 0(0.0) | 0.277 |
| **Glaucoma** | 2(8.3) | 2(2.7) | 5(1.8) | 1(3.1) | 0.263 |
| **Cataract** | 2(8.3) | 4(5.4) | 33(12.0) | 5(15.6) | 0.314 |
| **Pulmonary tuberculosis** | 3(12.5) | 3(4.1) | 19(6.9) | 1(3.1) | 0.415 |
| **COPD** | 2(8.3) | 15(20.3) | 37(13.5) | 3(9.4) | 0.299 |
| **Bronchiectasis** | 3(12.5) | 3(4.1) | 14(5.1) | 2(6.2) | 0.438 |
| **Chronic cough** | 3(12.5) | 4(5.4) | 30(10.9) | 9(28.1) | **0.009** |
| **Pneumonia** | 5(20.8) | 4(5.4) | 29(10.5) | 5(15.6) | 0.133 |
| **COVID-19** | 4(16.7) | 16(21.6) | 55(20.0) | 7(21.9) | 0.952 |

CCR, complete clinical remission; CR, clinical remission; PR, partial remission; NR, no remission; BMI, Body Mass Index; DM, diabetes; HTN, hypertension; MI, myocardial infarction; GERD, gastroesophageal reflux disease; COPD, chronic obstructive pulmonary disease; COVID-19, coronavirus disease 2019
